# Infectious diseases amidst a humanitarian crisis in Ukraine: A rising concern

**DOI:** 10.1016/j.amsu.2022.103950

**Published:** 2022-06-03

**Authors:** Mohammad Yasir Essar, Lolita Matiashova, Christos Tsagkaris, Valeriia Vladychuk, Michael Head

**Affiliations:** Kabul University of Medical Sciences, Kabul, Afghanistan; Clinical Informatics Research Unit, Faculty of Medicine, University of Southampton, Southampton, United Kingdom; T Malaya Therapy National Institute, National Academy of Medical Sciences of Ukraine, Kharkiv 61039, Ukraine; European Student Think Tank, Public Health and Policy Working Group, Amsterdam, Netherlands; Bogomolets National Medical University, Tarasa Shevchenko Blvd, 13, 01601, Kyiv, Ukraine; Clinical Informatics Research Unit, Faculty of Medicine, University of Southampton, Southampton, United Kingdom

**Keywords:** Infectious diseases, War, Healthcare, Ukraine

## Abstract

As of the 24th of February 2022, the war in Ukraine has increased the risk for infectious diseases outbreaks in the country and beyond. The disruption of healthcare services, the destruction of critical infrastructure, the displacement of millions of civilians and the crowded living conditions in bunkers pose a formiddable threat to public health. Infections are emphasized due to the low rates of vaccination against COVID-19 and the high prevalence of chronic infections such as Tuberculosis and HIV/AIDS in Ukraine compared to the WHO Europe region. Collaboration between authorities and humanitatian organizations is necessary, in order to strengthen epidemiological surveillance and deploy vital resources that are required for the prevention and the management of infections.

## Correspondence

As the hostility continues to escalate in Ukraine, the health needs of displaced and refugee Ukrainian populations will become increasingly urgent. Infectious disease outbreaks will be inevitable, and action to counter their surge is pivotal. Limited testing and vaccination capacity in combination with overcrowded settings (for example, in bunkers or refugee camps) can drive a COVID-19 outbreak. Other respiratory infectious diseases, including tuberculosis, influenza or measles, may spread easily in such settings, and control of other high-burden infections such as. AIDS will be more difficult [1]. Alongside urgent concerns such as blast injuries, additional infectious disease burdens may have a devastating effect on the fragile healthcare system of Ukraine and its populations [2].

Ukraine has a long and challenging history of infectious diseases. In 2018, Ukraine recorded around 30,000 new tuberculosis cases with 29% being drug-resistant [3]. In the WHO European Region, Ukraine has the fourth-highest TB incidence and fifth-highest number of confirmed cases of drug-resistant TB [4].

Active tracing of cases has reportedly been stopped in conflict–afflicted areas [5]. Access to diagnostic services and treatment is currently insecure. Delayed diagnosis and management may lead to poor treatment outcomes at individual level and continuous transmission of the infection in the community [5]. COVID-19 and TB pose a significant risk for public health in neighboring countries, where people from Ukraine seek refuge. UNAIDS estimated that there would be 1000–2000 Ukrainian refugees with tuberculosis [5].

Small-scale vaccine-derived polio outbreaks that have been observed before the conflict represent an additional source of concern. Two cases detected during 2020–2021 in combination with the isolation of poliovirus in 19 healthy contacts of the patients make the situation challenging. The conflict has required that an ongoing polio vaccination campaign, targeting 140,000 children, be paused. The Global Polio Eradication Initiative stressed that a lack of polio surveillance may lead to undetected outbreaks [6].

Measles outbreaks could surge in Ukraine, a common outcome from conflict settings where routine healthcare is hindered and vaccination campaigns stall, or stop altogether. [7]. A large and prolonged outbreak of >115,000 measles cases occurred in Ukraine across 2017–2020. [1].

Ukraine, bears the second highest burden of HIV/AIDS in Eastern Europe, with 1% prevalence across the general population, 21% prevalence in injecting-drug users and 7.5% in men who have sex with men [1]. Antiretroviral therapy (ART) has become scarce during the current crisis. High-risk behaviours, such as unsafe sex and risky drug injection practices, may also increase and thus increases the risk for new HIV infections [8].

## Conclusion

Overall, the unfolding crisis in Ukraine poses a risk for infectious outbreaks, and this is amidst the ongoing COVID-19 pandemic. The Ukraine health system is struggling to provide acute trauma care and also remain accessible for people in acute need of medical attention. All authorities and humanitarian organizations should continue monitoring the situation with displaced and refugee populations, and identify how best to target deployment of medicines, vaccinations and other vital resources. The military and political developments of the crisis may divert attention from health challenges; hence, it is crucial to ensure that infectious diseases are addressed, and do not become a forgotten burden of disease. The consequences of this would last for many years.

## Ethical approval

N/A.

## Source of funding

None.

## Author contribution

Mohammad Yasir Essar conceived the concept of the paper. Mohammad Yasir Essar, Lolita Matiashova, and Christos Tsagkaris wrote the first draft. Valeriia Vladychuk and Michael Head edited the second draft. All authors read and approved the final draft.

## Conflict of interest

None declared.

## Trail registry number

1. Name of the registry: N/A.

2. Unique Identifying number or registration ID: N/A.

3. Hyperlink to your specific registration (must be publicly accessible and will be checked): N/A.

## Guarantor

N/A.

## Consent

N/A.
